# Improvements for international medicine donations: a review of the World Health Organization Guidelines for Medicine Donations, 3rd edition

**DOI:** 10.1186/s40545-015-0045-3

**Published:** 2015-10-19

**Authors:** Nuria Cañigueral-Vila, Jennifer C. Chen, Lindsey Frenkel-Rorden, Richard Laing

**Affiliations:** MPH Health Policy and Management, Boston University School of Public Health, Boston, USA; MPH Epidemiology, Boston University School of Public Health, Boston, USA; MPH in Global Health, Boston University School of Public Health, Boston, USA; Department of Global Health, Boston University School of Public Health, Boston, USA

**Keywords:** Emergency, Medicine donations, Medicine donor, Medicine recipient, International development assistance, World Health Organization

## Abstract

Some humanitarian and development organizations respond to major natural disasters and emergencies by donating medicines. Many provide medicines on a routine basis to support health systems, particularly those run by Faith-Based Organizations. Although such donations can provide essential medicines to populations in great need, inappropriate donations also take place, with burdensome consequences. The World Health Organization (WHO) has developed the interagency *Guidelines for Medicine Donations* for use by donors and recipients in the context of emergency aid and international development assistance. Although comprehensive in nature and transferable to various emergency situations, adjustments to both content and formatting would improve this resource. Recommendations for the next version of these guidelines include: specific wording and consistent formatting; definition of who is a recipient, clear distinction between acute and long-term emergencies, and proper donation procedures pertaining to each; inclusion of visual aides such as flowcharts, checklists, and photos; and improving the citations system.

## Introduction

Large-scale emergencies dramatically affect survival and health of populations, making support from humanitarian and development organizations necessary for immediate relief. The response often involves the provision of medicines in the form of donations. While donating medicines may appear to be a simple and effective solution, the process is extremely complex and there can be significant undesirable consequences associated if the parties involved do not handle the process appropriately. Aiming to improve the medicine donation procedures, the World Health Organization (WHO), in cooperation with major international agencies active in humanitarian relief and development assistance, have formed guidelines for medicine donations [1]. The first edition of the guidelines was published in May 1996, later updated in 1999 and in 2010, producing the second and third editions, respectively.

This review analyzes the third edition of the *WHO Guidelines for Medicine Donations*. The objectives and target audience of the document are reviewed, and a summary of its content is provided. In addition, this review discusses the guidelines’ strengths and weaknesses, and provides suggestions for improvement. We hope these suggestions can be incorporated by the authors of the fourth edition.

## Review

### Objectives and target audience

The main objective of this manual is to provide a set of medicine donation guidelines that will guide recipients and donors through the donation process. The intention is to improve the quality of medicine donations in development and emergency aid. The target audience for this manual includes governments who wish to establish or revise national medicine donation guidelines, humanitarian agencies such as *Médecins Sans Frontières*, organizations who administer treatment in emergency situations such as Partners In Health, pharmaceutical companies, and organizations who manage donations. The guidelines emphasize that good medicine donation practices are of interest to donors as well as to recipients, and therefore organizations on both sides may wish to take advantage of its directions and recommendations.

## Summary

The Chapter 1 *Introduction* presents the objectives of the guidelines and describes the changes and improvements that have taken place since the previous two editions. The introduction also describes four core principles that form the basis of good donation practice regardless of the particular scenario, donation source, or beneficiary. In summary, these four principles are: (1) donations should benefit the recipient to the maximum extent possible; (2) donations should be given with due respect, and be in conformity with, the policies of the recipient; (3) there should be effective coordination between donor and recipient; and (4) there should be no double standard in quality.

The rationale for the guidelines is discussed in Chapter 2 *The need for guidelines*. Appropriate medicine donations have provided benefits; however, historically there have been examples of medicine donations that have caused serious negative outcomes. Some examples include the donation of excessive quantities of medicines during the 2001 earthquake in Gujarat, India [2], uncoordinated medicine donations after the Sri Lanka tsunami in 2004 [3], and perceived insufficient medicine quantities and lack of effective communication in Tanzania [4]. Problems associated with medicine donations can arise from inappropriate selection and quantity; lack of quality-ensured donors; inappropriate handling and management of donations; and product quality, especially related to expiry of short-dated medicines. These problems may incur costs to both donors and recipients, and can even have a negative impact on sustainable access to medicines [2–4].

Chapter 3 *Guidelines for medicine donations* describes twelve guidelines that are considered best practices for any medicine donation transaction. Guidelines are broken down by the following categories: selection of medicines; presentation, packaging, and labeling; and information and management. Guidelines are followed by a justification and explanation statement, as well as possible exceptions.

The roles and responsibilities for donors and recipients are summarized in Chapter 4 *Guidance to donors and recipients.* This chapter notes the importance of establishing expectations, monitoring and evaluation plans, and rules of phasing out long-term donations.

Lastly, Annex 1 *Examples of problems with medicine donations* summarizes three case studies where donations were not properly made. Reference is made throughout the chapters more specifically to the case from Sri Lanka after the tsunami in 2004, where the occurrence of inappropriate medicine donations caused significantly more harm to the relief efforts than good.

While donors may have good intentions, potential inconveniences to the recipient country are not always considered, which solidifies the need for clear guidelines. The first two chapters of the reviewed guidelines emphasize the importance of getting both donors and recipients involved and accountable in the process of donating medicines while the last two listed specific guidelines and responsibilities of the donor and recipient. These four chapters with the summaries of three case studies make up the *WHO Guidelines for Medicine Donations* 3rd edition.

### Strengths

The manual provides a comprehensive yet concise set of guidelines aimed at ensuring appropriate medicines donation procedures. The concepts are transferable to different types and sizes of donations, as well as varying country contexts. The manual is broken up into digestible sections, all of which provide reasonable and relevant recommendations to employ. Each of the twelve guidelines’ main statement is contained in a shaded box, which keeps the text organized and makes it easy to follow.

Annex 1 provides a satisfactory overview of some key case studies where medicine donations had unfavorable outcomes, which serve as reminders of how important good donation practices are in emergency situations. It also demonstrates the necessity for explicit national donation guidelines. For example, guideline #6 states:

*“After arrival in the recipient country all donated medicines should have a remaining shelf-life of at least one year. […] all donated quantities should match the needs to be consumed before they are expired.”*

On the contrary, we saw in the Sri Lanka case study that 50 % of medicine donations to Sri Lanka did not have an expiry date and 6.5 % were expired on arrival to the country sites. Not only were these medicines unusable, but they also caused extra operational costs, thus aggravating already bad conditions in the recipient country and raising questions about the intentions of such donations. Additionally, 67 % of medicines Sri Lanka received with a shelf life of less than a year were likely to have expired long before their actual use. Inclusion of this case study illustrates the importance of observing the guidelines to manage medicine donations appropriately especially in emergency situations. In the next edition of the guidelines, the authors may want to refer to more recent cases, such as those described in *Additional resources, A.*

### Suggestions for improvements

Although some elements of this resource are intentionally broad to allow for situational tailoring, the lack of definition of who is a “recipient” limits the benefits of this resource. By including a broad definition of appropriate recipients, such as governments, NGOs, and health facilities, the benefits of medicine donations could be enhanced. This is especially relevant in scenarios where a government is unstable or there is a severe lack of capacity, and NGOs and/or health facilities are in a position to take on the role of managing a donation. Legitimate concerns have been expressed about the power of governments to control the flow of donations [5], and thus defining legitimate medicine donation recipients upfront may be a helpful way to emphasize an inclusive process. This could be included at the beginning of Chapter 2*,* following the mention of different medicine donation scenarios.

While most of the guidelines and associated explanations are clear, concise, and leave room for the local context to be considered, several guidelines in Chapter 3 are vague. For example, the third guideline states: “The presentation, strength, and formulation of donated medicines should, as far as possible, be similar to those of medicines commonly used in the recipient country.” This guideline could be rephrased to state that donated medicines should be provided in formulations that are in accordance with national regulations and treatment guidelines since “as far as possible” and “similar” is open to a wide variety of interpretations. Although there is need for flexibility in the application of the guidelines to accommodate the local context, there should be no exceptions for mislabeled or wrongly labeled products; therefore, a sub-section under ‘possible exceptions’ should emphasize ‘no exceptions’ in an effort to keep a consistent format with other guidelines.

On the other hand, emergency situations may vary widely in nature, and medicine donation guidelines should reflect the need to tailor procedures accordingly. Although reference is made to both acute emergencies and long-term scenarios, he guidelines are explicitly generalized, blending roles and responsibilities, which may ultimately confuse the user. Therefore, Chapter 3 should clearly state under each guideline considerations for acute emergencies (such as high-level armed conflicts or sudden natural disasters) versus long-term complex scenarios (such as persistent low-level armed conflicts, refugee communities, or ongoing development aid) as required, or otherwise indicate the same procedure may be used regardless of the nature of the emergency. For example, certain exceptions that might be necessary in acute emergencies due to urgency for a solution may be inappropriate in the context of long-term scenarios. Adding a table separately listing examples of both types of emergencies and/or including case studies of successful organizations’ donations protocols would further aid users in discerning the most appropriate procedures to follow in a given case. The authors could learn from the long-term donation programs set by organizations such as the Catholic Medical Mission Board [6] and Eli Lilly [7, 8].

In Chapter 4 *Guidance to donors and recipients*, we noticed that many of the surrounding issues were already touched upon in Chapter 3. The difference is that Chapter 4 is separated by the different burdens of responsibility. For instance, Chapter 4 states that medicine donations from donors should be guided by needs, and recipients should define the extent of their need, similar to what Chapter 3 guideline #1 states. It should be made clearer that Chapter 3 encompasses guidelines for donations while Chapter 4 explicitly differentiates the roles and responsibilities donors and recipients play in each process. Similar to the *WHO Guidelines for health care equipment donations* (see *Additional resources, B*), process flowcharts could be included for a clear visualization of each party’s responsibilities and interdependencies. Similarly, checklists for both donors and recipients at different stages of the donation process would improve understanding as well as make this tool readily available for use in a real emergency.

Although cash donations are usually preferred to product donations, we suggest the guidelines explain some limitations of cash donations under sub-section 4.2 *Guidance for donors*. This would provide a more well-rounded understanding of the various implications of donating money to organizations and governments as cash does not always translate into aid, let alone medicines, in the context of an emergency. For example, the recipient may lack capacity to receive and manage money [9, 10] and donors may not act transparently when managing cash donations [11].

As a whole, the manual provides a clear macro-level picture of the aspects donors and recipients should take into account regarding medicine donations procedures but falls short in providing action-oriented, practical advice, and recommendations at the micro-level. Therefore, an organization receiving the guidelines may find it difficult to do anything more than understand the importance of maintaining good medicine donation practices. More detailed steps are necessary to actually carry out a medicine donation in an appropriate fashion. In addition to including process flowcharts and checklists in Chapter 4, suggested timelines for donation procedures should accompany them in an effort to advise and guide governments and/or organizations who are new to donating medicines. We suggest future authors of the *WHO Guidelines for Medicine Donations* 4th edition contact various organizations who have significant experience making donations to develop realistic time estimates around operational procedures.

Additionally, including visual imagery throughout the manual would not only break up the text and accelerate absorption of content from the reader’s perspective, but could also make the information more compelling for existing and new actors in the medicine donation environment, potentially increasing appropriate practice utilization. The next edition of the guidelines should consider the inclusion of relevant pictures illustrating the problems that justify each of the presented guidelines. For instance, including photos of counterfeit labeling could be helpful examples in section 3.2 *Presentation, packaging, and labeling.* Illustrations of expired drug stockpiles and medicine destruction could be included in either section 3.1 *Selection of medicines* or section 3.3 *Information and management*. Other images could show the human labor required to manage the storage of donated medicines and other striking pictures for visualization and context.

With regard to the formatting of the manual, the way footnotes and references are currently cited within the text is confusing. Numbers are used for both footnotes and references. For example, number 1 is used several times in the text to reference footnotes on different pages, and simultaneously used for the first reference provided at the end of the guidelines. This may be improved by changing the footnote coding to alphabet characters. Additionally, more attention should be paid when referencing boxes within the text, as section 4.1 (page 12 in the reviewed WHO guidelines [1]) incorrectly refers to Box 1 instead of Box 2. Maintaining consistent and accurate labeling and referencing will make the manual more clear to readers.

Lastly, adding an Annex that includes a list of organizations that have referenced this manual when donating medicines in the past would be helpful for others who wish to use these guidelines. This would add credibility to the manual and may motivate further use.

## Conclusions

Medicine donation practices that have occurred previously during natural disasters and other emergency situations expose both the positive and detrimental effects donations can have on a recipient country. Past experiences show that guidelines for the donation of medicines are both necessary and crucial to promoting efficient distribution, handling, and use of such products. The principles outlined in the WHO *Guidelines for Medicine Donations* are clear and concise, yet not particularly practical due to the omission of step-by-step, action-oriented instructions. There is room for improvement from 2 perspectives: (1) from the user’s perspective, these guidelines could include additional tools (e.g. flowcharts, checklists, and pictures) to facilitate the donation process engaged in by donors and recipients; (2) from the reader's perspective, the guidelines’ structure could be reorganized to improve understanding and make information more digestible. Such adjustments could improve guideline adoptability by all involved parties and optimize donation procedures with the potential to improve outcomes in the future.

## Additional resources for a fourth edition

A. Bero L, Carson B, Moller H, Hill S. To give is better than to receive: compliance with WHO guidelines for drug donations during 2000–2008. Bull World Health Organ. 2010 Dec 1; 88(12): 922–929. http://www.ncbi.nlm.nih.gov/pmc/articles/PMC2995193/pdf/BLT.10.079764.pdf. *This article assesses diverse drug donations in terms of adherence to the WHO Guidelines for Medicine Donations. It is a good source of case studies that could be used to update or expand Annex 1 in the WHO guidelines with more recent situations.*
B. World Health Organization (WHO): Guidelines for health care equipment donations. 2000. http://www.who.int/medical_devices/publications/en/Donation_Guidelines.pdf. Accessed 2015 Feb 1. *Besides outlining the principles of good donation practices, this document stresses the importance of recipient policies, donor coordination, and the necessity for planned health care equipment donations. Figures 1-5 in pages 3-8 provide good examples of process flowcharts and checklists used to plan for medical equipment donations. These could serve as a model in the effort to make a more action-oriented, practical next version of the reviewed WHO Guidelines for Medicine Donations.*
